# Effect of Dual Antiplatelet Therapy on Shunt Outcomes in Patients with Aneurysmal Subarachnoid Hemorrhage: A Matched Cohort Pilot Study

**DOI:** 10.7759/cureus.2383

**Published:** 2018-03-28

**Authors:** Gabriella M Paisan, Dale Ding, Zhiyuan Xu, Kenneth C Liu

**Affiliations:** 1 Department of Neurological Surgery, University of Virginia, Charlottesville, USA; 2 Neurosurgery, Barrow Neurological Institute, St. Joseph's Hospital and Medical Center, Phoenix, AZ, USA

**Keywords:** dual antiplatelet therapy, hydrocephalus, intracranial aneurysm, shunt, stent, subarachnoid hemorrhage

## Abstract

Objective: The aim of this retrospective, matched cohort study is to determine the effect of dual antiplatelet therapy (DAPT) on shunt-related complications and long-term functional outcomes in endovascularly treated aneurysmal subarachnoid hemorrhage (aSAH) patients.

Materials and Method: We retrospectively analyzed an institutional database of aSAH patients from 2000-2015. Patients who underwent endovascular treatment with stent-assisted coiling (DAPT cohort) were matched in a 1:4 ratio to those who underwent coiling alone (no-DAPT cohort) based on the presenting patient and aneurysm factors. A favorable outcome was defined as a modified Rankin scale of <2. Statistical analyses were performed to compare the shunt-related and functional outcomes between the DAPT and no-DAPT cohorts.

Results: After applying the selection criteria and performing the matching process, the overall study cohort comprised 25 aSAH patients who underwent endovascular treatment, including five in the DAPT and 20 in the no-DAPT cohorts. The mean age, World Federation of Neurological Surgeons grade, aneurysm size, and follow-up duration of the overall study cohort were 52.3 years, 2.9, 7.4 mm, and 32.7 months, respectively. The mean time from aSAH to shunt placement was significantly higher for patients in the DAPT cohort (5.6 vs. 0.7 months; p=0.026). The shunt complication rates (p=0.562) and functional outcomes at last follow-up (p=0.924) were not significantly different between the two cohorts.

Conclusion: Patients receiving DAPT after the stent-assisted coiling of acutely ruptured aneurysms do not have an increased risk of shunt-related complications or unfavorable long-term functional outcomes compared to endovascularly treated aSAH patients not taking DAPT. These results suggest that further study is warranted.

## Introduction

Chronic hydrocephalus is one of the most common sequelae of aneurysmal subarachnoid hemorrhage (aSAH) and has been reported to occur in 9%-36% of ruptured aneurysm patients [1-5]. Endovascular therapy is currently the preferred intervention for ruptured aneurysms at many centers, but large (diameter >10 mm) and wide-necked (dome-to-neck ratio <2 mm or neck width >4 mm) aneurysms are challenging to successfully treat with coiling alone [6-11]. In order to safely target this subset of anatomically unfavorable aneurysms from an endovascular approach, stents have emerged as an efficacious adjunctive device [12-15]. However, neurointerventionalists may be wary of performing stent-assisted coiling in the setting of aSAH since the necessity of postoperative dual antiplatelet therapy (DAPT) to prevent thromboembolic events increases the subsequent risk of hemorrhagic complications after surgical procedures, such as cerebrospinal fluid (CSF) shunting. Although prior studies have reported an increased periprocedural risk of intracranial hemorrhage after the ventriculostomy insertion or ventriculoperitoneal shunt placement in patients on DAPT, there remains an overall paucity of data regarding the clinical implications of DAPT on CSF diversion [16-18]. Therefore, the aim of this retrospective matched cohort study is to determine the effect of DAPT on shunt-related complications and long-term functional outcomes in aSAH patients who underwent endovascular intervention and subsequently developed shunt-dependent hydrocephalus.

## Materials and methods

Study design

With IRB approval (IRB number 17082), we retrospectively evaluated a database consisting of all spontaneous SAH patients who were treated at the University of Virginia Health System from 2000-2015. Criteria for inclusion in the study were as follows: (1) spontaneous SAH secondary to a ruptured intracranial aneurysm, (2) endovascular treatment of the ruptured aneurysm, (3) chronic hydrocephalus after aSAH requiring permanent CSF diversion, (4) available baseline data regarding the patient and aneurysm characteristics at presentation, and (5) available follow-up data, specifically with regard to functional outcomes and shunt complications.

For this analysis, patients were divided into the DAPT and no-DAPT cohorts. All patients in the DAPT cohort underwent endovascular treatment of their ruptured aneurysms with stent-assisted coiling, whereas all patients in the no-DAPT cohort underwent treatment with coiling alone. The standard DAPT regimen used at our institution is aspirin 325 mg and clopidogrel 75 mg daily. Patients who were treated with coiling alone but were administered antiplatelet agents or anticoagulated postoperatively (e.g., for intraprocedural thromboembolic complications or coil herniation into the parent artery) were excluded. During the acute hospitalization, we shunted patients who were unable to be weaned off of their external ventricular drains (EVDs). We also shunted patients in a delayed setting who had interval ventricular enlargement, consistent with hydrocephalus, with or without associated neurological symptoms. The patients were maintained on the same DAPT regimen before and after the shunt procedure, without any planned cessation of either antiplatelet medication. All patients underwent a routine postoperative brain computed tomography (CT) within four hours of shunt placement to evaluate for radiographic complications, and they were assessed with serial neurological exams to evaluate for clinical complications. 

Baseline data and follow-up

The following demographic, aneurysm, treatment, and hospitalization variables were determined for each patient selected for the study cohort: patient age and gender, presence of acute hydrocephalus at presentation, and necessity for external ventricular drain (EVD) placement - Fisher, Hunt and Hess, and World Federation of Neurological Surgeons (WFNS) grades; aneurysm location (classified as anterior vs. posterior circulation) and size (maximum diameter), endovascular treatment method (classified as stent-assistance or coiling alone), use of DAPT, occurrence of angiographic cerebral vasospasm, shunt type (categorized as ventriculoperitoneal, ventriculopleural, or lumboperitoneal), and time from aSAH to shunt placement [19-21].

Patients were evaluated for functional outcome at the most recent follow-up using the modified Rankin Scale (mRS); favorable outcome was defined as an mRS of <2 (functional independence), whereas unfavorable outcome was defined as an mRS of >3 (functional dependence or death) [22]. Patients were also assessed for shunt revisions and complications. Shunt complications included shunt infection, inadequate placement of proximal or distal catheters requiring revision, inadequate shunting due to valve malfunction, intraventricular hemorrhage, intraparenchymal hemorrhage along the shunt tract, development of a CSF pseudocyst, wound breakdown, and exposed hardware.

Statistical analysis

Data were presented as mean and standard deviation (SD) for continuous variables and as frequencies for categorical variables. Patients in the DAPT cohort were matched to those in the no-DAPT cohort, in a 1:4 ratio, based on the following factors: patient age and sex; acute hydrocephalus and EVD placement; Fisher, Hunt and Hess, and WFNS grades; and aneurysm location (stratified as anterior vs. posterior); and size. Continuous variables were compared using the unpaired student’s t-test. Categorical variables were compared using Fisher’s exact test. All statistical tests were two-sided and statistical significance was defined as P<0.05. All statistical analyses were calculated using SPSS.

## Results

Baseline characteristics of the DAPT vs. no-DAPT cohorts

Of the 116 aSAH patients who developed shunt-dependent hydrocephalus, 49 (42.2%) underwent endovascular aneurysm treatment. Stent-assisted coiling was used to treat five patients, all of whom were administered DAPT postoperatively. Coiling alone was used to treat 44 patients. After the 1:4 matching process, a total of 25 patients were selected for analysis in this study, including five in the DAPT and 20 in the no-DAPT cohorts.

The mean age of the overall study cohort was 52.3±13.0 years, and it consisted of 20 females (80%). The Fisher grade was four for all patients, and the mean Hunt and Hess grade was 3.2±1.1. The mean Glasgow Coma Scale (GCS) at presentation and World Federation of Neurosurgeons (WFNS) grade were 11.2±3.7 and 2.9±1.5, respectively. Acute hydrocephalus was present in 24 patients (96%), and 22 underwent EVD placement (88%). Nine aneurysms were located in the posterior circulation (36%), and the mean aneurysm size was 7.4±5.5 mm.

Table 1 compares the patient and aneurysm characteristics of the matched DAPT and no-DAPT cohorts. There were no significant differences in any of the matched factors. The mean aneurysm size of the DAPT cohort was larger (11.0 vs. 6.5 mm), but this difference was not statistically significant (p=0.108).

**Table 1:** Comparison of patient and aneurysm characteristics of the matched DAPT and no-DAPT cohorts. *Statistically significant (P<0.05); SD=standard deviation; EVD=external ventricular drain; GCS = Glasgow Coma Scale; WFNS = World Federation of Neurosurgeons

Factor	DAPT (N=5)	No-DAPT (N=20)	P-Value
Age (mean±SD years)	52.2±15.6	52.3±12.7	1.00
Female sex	4 (80%)	16 (80%)	1.00
EVD placement	4 (80%)	18 (90%)	0.504
Posterior circulation aneurysm location	3 (60%)	6 (30%)	0.312
Aneurysm size (mean±SD mm)	11.0±8.0	6.5±4.6	0.108
Acute hydrocephalus	5 (100%)	19 (95%)	1.00
Fisher grade	4±0	4±0	1.00
Hunt and Hess grade	3.4±0.5	3.2±1.1	0.712
GCS at presentation	11.0±2.8	11.3±3.9	0.896
WFNS grade	3.4±0.9	2.8±1.6	0.435

Outcomes of the DAPT vs. no-DAPT cohorts

Table 2 compares the outcomes of the DAPT and no-DAPT cohorts. Patients in the DAPT cohort had significantly longer time intervals from presentation with aSAH to shunt placement (mean 5.6 vs. 0.7 months; p=0.026). There were no other differences in outcomes between the DAPT and no-DAPT cohorts. Although the shunt complication rate of the DAPT cohort was higher (40% vs. 20%), this difference was not statistically significant (p=0.562).

**Table 2:** Comparison of the outcomes of the DAPT and no-DAPT cohorts *Statistically significant (P<0.05); SD=standard deviation; *Unfavorable outcome=mRS 3-6 (functional dependence or death) DAPT: dual antiplatelet therapy

Factor	DAPT (N=5)	No-DAPT (N=20)	P-Value
Shunt Complications	2 (40%)	4 (20%)	0.562
New Postoperative Intraventricular Hemorrhage	1 (20%)	1 (5%)	0.367
Shunt Revision	1 (20%)	7 (35%)	0.642
Shunt Infection	0 (0%)	2 (10%)	1.00
CSF Pseudocyst	1 (20%)	0 (0%)	0.200
Wound Breakdown	0 (0%)	2 (10%)	1.000
Valve Malfunction	1 (20%)	1 (5%)	0.367
Angiographic Vasospasm	4 (80%)	17 (85%)	1.00
Time Interval from aSAH to Shunt Placement (mean±SD months)	5.6±9.8	0.7±0.9	0.026*
Follow-up Duration (mean±SD months)	30.4±24.7	33.3±34.1	0.860
mRS at Last Follow-Up	2.0±1.7	2.1±2.1	0.924
Unfavorable Outcome* at Last Follow-Up	1 (20%)	7 (35%)	0.210

The mean follow-up time of the overall study cohort was 32.7±32.0 months, which was not significantly different between the DAPT and no-DAPT cohorts (mean 30.4 vs. 33.3 months, respectively; p=0.860). There was no statistically significant difference between the mean mRS at last follow-up between the DAPT and no-DAPT cohorts (2.0 vs. 2.1; p=0.924), and the rates of unfavorable outcome (mRS 3-6) were also not significantly different (DAPT cohort 20% vs. no-DAPT cohort 35%; p=0.210).

## Discussion

Key findings

Stent-assisted coiling has expanded the endovascular repertoire for aneurysm treatment and is particularly useful for large or wide-necked lesions that are not amenable to coiling alone [23-24]. In order to mitigate the risk of thromboembolic complications, patients who undergo stent-assisted coiling are routinely administered DAPT postoperatively. The requirement for DAPT in stent-coiled aSAH patients evokes a precarious hospital course, as one must balance the risk of intracranial and systemic hemorrhagic complications associated with antiplatelet administration against the risk of stent thrombosis and ischemic stroke associated with a cessation of DAPT. Due to these concerns, some cerebrovascular surgeons prefer to surgically clip ruptured aneurysms, which cannot be readily secured with coiling alone [25]. Alternatively, neurointerventionalists may opt to use adjunctive endovascular techniques not requiring DAPT, such as balloon remodeling or the dual microcatheter technique [11,26]. Nevertheless, depending on the preferences and experience of the treating neurointerventionalist, stent-assisted coiling has been shown to afford a reasonable risk to the benefit profile for appropriately selected ruptured aneurysms [12–15].

In our matched cohort analysis, we did not find a significant difference between the shunt complications rates of the DAPT vs. no-DAPT cohorts. This contradicts similar analyses performed in prior studies, although one should note that comparatively, the size of our DAPT cohort (N=5) is relatively small. Tumialán et al. assessed the outcomes after EVD or shunt placement in seven patients on DAPT after stent-assisted aneurysm coiling and reported an 86% intracranial hemorrhage rate [18]. Kung et al. compared the hemorrhage rates after EVD or shunt placement between endovascularly treated aSAH patients assigned to DAPT and no-DAPT cohorts and reported significantly higher rates of both radiographic (32% vs. 15%; p=0.02) and symptomatic hemorrhage (8% vs. 1%; p=0.03) in the DAPT cohort [17]. Mahaney et al. also evaluated the effect of DAPT on hemorrhagic complications after shunt placement in aSAH patients, and found a significantly higher hemorrhage rate in the DAPT cohort (33% vs 0%; p=0.0075), although only one hemorrhage was clinically significant and no patients suffered permanent morbidity secondary to a shunt-related hemorrhage [16].

The pathophysiology of hydrocephalus after aSAH is complex and multifactorial. As such, the causes of the development of hydrocephalus acutely after aSAH versus weeks or months after aSAH maybe distinct. In our analysis, patients in the DAPT and no-DAPT cohorts had significantly different time internals to shunt placement after aSAH (5.6 vs. 0.7 months; p=0.026). Therefore, it is possible that this suggests that this cohort's post-aSAH hydrocephalus is of a different etiology than the no-DAPT cohort. However, we believe that this difference is more likely due to the reluctance of practitioners to perform shunting in the setting of DAPT. Additionally, we found no difference in long-term functional outcomes between the cohorts. Therefore, although the mechanistic differences between acute and late-onset post-aSAH hydrocephalus are deserving of future study, it remains unclear if the underlying differences in pathophysiology represent differential responses to treatment or portend different clinical courses.

In contrast to the previous studies of DAPT in aSAH patients with shunt-dependent hydrocephalus, ours is the first to utilize a matched cohort design to account for the differences in baseline patient and aneurysm factors between those treated with stent-assisted coiling vs. coiling alone. Furthermore, we present the first analysis of the effect of DAPT on long-term functional outcomes after aSAH. Despite the potentially harmful consequences of hemorrhagic complications on DAPT, we found that the long-term functional outcomes (mean overall follow-up 33 months) were similar between the DAPT and no-DAPT cohorts. Therefore, it is possible that although aSAH patients on DAPT could incur an elevated risk of shunt-related hemorrhage, as noted in prior studies, these hemorrhages may not impart enough neurological injury to adversely affect long-term functional outcomes [16–18]. However, we caution that our results should not be taken to imply that DAPT can be administered with impunity in aSAH patients. The relationship among DAPT use, shunt complications, and functional outcomes represents only a component of the overall management of aSAH patients, which is multifactorial and complex. A large, multicenter cohort study of shunt outcomes in aSAH patients on DAPT is warranted to clarify the management of this challenging subset of patients. Additionally, further studies in larger patient cohorts are necessary to determine risk factors for complications and unfavorable outcomes in aSAH patients on DAPT who undergo CSF shunting.

Limitations and generalizability

The validity of our findings is limited by numerous factors, the most notable of which is the small size of the DAPT cohort, such that our statistical analysis was insufficiently powered to detect a significant difference in shunt complications and functional outcomes between the two cohorts. Additionally, the single-center, retrospective design of our study subjects our analyses to the selection, treatment, and referral biases of our institution and its treating physicians. Finally, none of the patients in the DAPT cohort were treated with a flow-diverting stent, and since the conventional stents and flow diverters have distinct physical properties and safety profiles, our findings cannot be generalized to ruptured aneurysm patients treated acutely with flow diversion [27-30].

## Conclusions

We failed to find an increased risk of shunt-related complications or worse functional outcomes in patients who were administered DAPT after acute intervention for a ruptured aneurysm with stent-assisted coiling compared to those who underwent coiling alone and did not receive DAPT. Although further analyses of larger patient cohorts are necessary for validation, our findings suggest that the specter of poorer shunt outcomes in the setting of DAPT use may be unwarranted and therefore should not deter the employment of stent-assisted coiling for the acute treatment of appropriately selected patients with ruptured aneurysms. The significantly longer time interval between presentation with aSAH and shunt placement in the DAPT cohort may reflect the reluctance of practitioners to perform surgical procedures on this subset of patients.
